# Supplementary material for: A continuous record of early human stone tool production
Source: Camb Prism Extinct. 2025 Dec 19;4:e1. doi: 10.1017/ext.2025.10009 (PMC12800537; doi:10.1017/ext.2025.10009)
Supplement: Key and Williams supplementary material [file S2755095825100090sup001.docx]

**Supplementary Information**

In addition to investigating cultural cohesion across the four largest gaps in the Oldowan record we also ran the surprise test, as described in the main methods, across all other temporal gaps in the site sample (minus those removed due to age-range overlap and close proximity [again, following the main methods]). This resulted in a further 34 temporal gaps being investigated, as detailed in Supplementary Tables 1 and 2. A *k* of 5 was used in all instances for these additional tests, with forwards and backwards models run when possible, using central age data only (i.e., the resampling approach was not used for these additional analyses). Supplementary Table 2 presents the results of these tests. No significant results were returned when α = .05, with p ≥ .3421 in all instances. Therefore, consistent with the main analyses, no temporal gaps were large enough on a relative basis to infer a loss of cultural information. The null hypothesis that all occurrences were produced by the same cultural process is therefore accepted, and there is no temporal evidence for a loss of stone tool making knowledge by Oldowan hominins.

As neither of our approaches account for the date ranges independently attached to an Oldowan occurrence’s upper or lower date-range limit, it is useful to highlight that our resampling approach and the widespread use of palaeomagnetism dating means this consideration likely had minimal impact on the results. Principally, this is because a large proportion of the upper/lower date ranges attached to Oldowan occurrences are defined by well-dated (i.e., with minimal error) palaeomagnetic reversals. The second reason is that our resampling approach rarely samples dates from the temporal periods close to an occurrence’s upper/lower limits. Our use of a normal distribution means these extremes would be infrequently sampled and error ranges at these temporal points would have minimal impact on the results.

Given the surprise test assumes a Gumbel distribution we tested whether the central ages in Table 1 fitted an alternative, exponential distribution using the maximum likelihood method from the fitdistrplus package in R, using rescaled data (divided by 1,000,000). A Komogorov-Smirnov test returned a *p* value of <0.0001 (D = 0.5689), confirming the Oldowan date data to not come from an exponential distribution. Consequently, when compared against a Gumbel distribution (using the same rescaled data, resulting in a mode of 2.11 and spread of 0.2340), a null of no difference cannot be rejected following a Komogorov-Smirnov test (p = 0.7065, D = 0.1372). Additionally, we tested whether the Oldowan record fitted a Weibull distribution, again using the fitdistrplus package and rescaled data. We failed to reject a Weibull distribution via. a Komogorov-Smirnov test (p = 0.5049, D = 0.1587), further strengthening our inference of a continuous record subsequent to the start of the Oldowan (i.e., there is a growth in site frequency through time which fits a Weibull curve, as expected when a cultural trait originates, spreads, and becomes increasingly engrained within a species’/populations behaviour). Thus, an assumed Gumbel distribution is appropriate, with the deviation from exponential likely being due to the increased rate of Oldowan artefacts entering the archaeological record in later time periods (due to increased dependence by hominins, larger populations using the tools, or taphonomic considerations).

**Supplementary Table 1:** The 39 Oldowan occurrences used in the supplementary analyses, their temporal data, and references for where these data were procured. ‘Test rank’ refers to the age ranking of sites after those with localised (<10 km) date-range overlap were removed, while ‘site rank’ refers to a site’s age ranking within the complete sample of 91 Oldowan occurrences.

| **Test Rank** | **Site Rank** | **Oldowan Site** | **Central Date BP** | **Date Range BP** | **References** |
| --- | --- | --- | --- | --- | --- |
| 1 | 1 | Nyayanga NY-1 | 2900000 | 3032000 - 2595000 | Plummer et al., 2023 |
| 2 | 2 | Namorotukunan-1 | 2750000 | 3440000 – 2610000 | Braun et al., 2025 |
| 3 | 3 | Ledi-Geraru BD 1 | 2581000 | 2610000 - 2581000 | Braun et al., 2019 |
| 4 | 4 | Namorotukunan-2 | 2580000 | 2610000 - 2137000 | Braun et al., 2025 |
| 5 | 13 | Gona OGS-7 | 2565000 | 2580000 - 2550000 | Semaw et al., 2003 |
| 6 | `5 | Ain Boucherit AB-Lw | 2440000 | 2580000 - 2300000 | Sahnouni et al., 2018 |
| 7 | 17 | Hadar A.L. 666 | 2352500 | 2360000 - 2330000 | Kimbel et al., 1996 |
| 8 | 18 | Lokalalei LA1A | 2330000 | 2390000 - 2290000 | Tiercelin et al., 2010 |
| 9 | 24 | Omo Shungura FtJi 1-3-4 | 2329000 | 2334000 - 2324000 | McDougall and Brown, 2008 |
| 10 | 27 | Omo Shungura 1/E-2 | 2297000 | 2324000 - 2270000 | Maurin et al., 2017 |
| 11 | 28 | Nasura NAS2 | 2270000 | na | Boës et al., 2024 |
| 12 | 29 | Lokalalei 2C (+2A, 2D) | 2266000 | 2390000 - 2266000 | Tiercelin et al., 2010 |
| 13 | 31 | Nasura NAS3 | 2250000 | na | Boës et al., 2024 |
| 14 | 31 | Swartkrans Member 1 | 2220000 | 2310000 - 2130000 | Kuman et al., 2021 |
| 15 | 34 | Gona DAN-2 | 2200000 | 2400000 - 2000000 | Stout et al., 2005 |
| 16 | 35 | Sterkfontein Member 5 | 2180000 | 2390000 - 1970000 | Granger et al., 2015 |
| 17 | 36 | Kanjera South ( KS1-3) | 2000000 | 2300000 - 1920000 | Ditchfield et al., 2019 |
| 18 | 37 | Olduvai Gorge L.68 | 1995500 | 2015000 - 1976000 | Stollhofen et al., 2021 |
| 19 | 38 | Drimolen Main Quarry | 1995000 | 2040000 - 1950000 | Stammers et al., 2018 |
| 20 | 40 | Gona OGS-3 | 1950000 | 2200000 - 1700000 | Rogers et al., 2023 |
| 21 | 41 | Ileret FwJj 20 (A 41) | 1950000 | na | Braun et al., 2010 |
| 22 | 42 | Fejej FJ-1a | 1950000 | 1980000 - 1920000 | Barsky et al., 2011 |
| 23 | 43 | Ain Boucherit Ab-Up | 1920000 | 1970000 - 1850000 | Sahnouni et al., 2018 |
| 24 | 44 | Konso KGA6-A1 | 1910000 | 1940000 - 1880000 | Beyene et al., 2013 |
| 25 | 45 | Kromdraai B | 1885000 | 2000000 - 1770000 | Braga and Thackerary 2016 |
| 26 | 48 | Koobi Fora FxJj1 105 | 1880000 | 1900000 - 1640000 | Isaac et al., 1997 |
| 27 | 49 | Olduvai Gorge DK | 1864000 | 1880000 - 1848000 | Proffitt, 2018 |
| 28 | 53 | Wonderwerk Stratum 12 | 1850000 | 1930000 - 1770000 | Shaar et al., 2021 |
| 29 | 59 | Olduvai Gorge FLK Zinj | 1843500 | 1848000 - 1839000 | Proffitt, 2018 |
| 30 | 61 | Kokiselei KS 5,6 | 1800000 | 1870000 - 1650000 | Texier, 2018 |
| 31 | 62 | Kilombe GqJh13A,12B | 1780000 | 1800000 - 1700000 | Gowlett et al., 2022 |
| 32 | 64 | El Beidha Levels A-C | 1770000 | 1800000 - 1740000 | Duval et al., 2021 |
| 33 | 67 | Naiyena Engol NY1 | 1750000 | 1800000 - 1700000 | Roche et al., 2018 |
| 34 | 68 | Kalokodo 6 | 1750000 | 1800000 - 1700000 | Roche et al., 2018 |
| 35 | 69 | Olduvai Gorge HWK E | 1741500 | 1803000 - 1680000 | de la Torre and Mora, 2018 |
| 36 | 72 | Melka Kun. Garba IVE,IVF | 1719000 | 1719000 - 1719000 | Gallotti and Mussi, 2015 |
| 37 | 74 | Ain Hanech Levels A-C | 1700000 | 1700000 - 1700000 | Parés et al., 2014 |
| 38 | 77 | Sare-Abururu SR-2 | 1700000 | 1775000 - 1567000 | Finestone et al., 2024 |
| 39 | 79 | Melka Kun. Kella III | 1666000 | 1666000 - 1260000 | Morgan et al., 2012 |

**Supplementary Table 2:** Significance values using Solow and Smith's (2005) surprise test when applied to all temporal breaks visible in the Oldowan archaeological (*α* = .05). ‘Same central age’ is noted when the central age of the two sites compared are the same.

| **Temporal Gap Investigated** | ***p*** | |
| --- | --- | --- |
|  | **Forwards Model** | **Reverse Model** |
| Nyayanga NY-1 ↔ Namorotukunan 1 | In the main analysis | |
| Namorotukunan 1 ↔ Ledi-Geraru BD 1 | In the main analysis | |
| Ledi-Geraru BD 1 ↔ Namorotukunan 2 | - | .9954 |
| Namorotukunan 2 ↔ Gona OGS-7 | - | .9070 |
| Gona OGS-7 ↔ Ain Boucherit AB-Lw | .7198 | .3421 |
| Ain Boucherit AB-Lw ↔ Hadar A.L. 666 | In the main analysis | |
| Hadar A.L. 666 ↔ Lokalalei LA1A | .8661 | .6841 |
| Lokalalei LA1A ↔ Omo Shungura FtJi 1-3-4 | .9954 | .9836 |
| Omo Shungura FtJi 1-3-4 ↔ Omo Shungura 1/E-2 | .8863 | .6487 |
| Omo Shungura 1/E-2 ↔ Nasura NAS2 | .8372 | .6884 |
| Nasura NAS2 ↔ Lokalalei 2C (+2A, 2D) | .9421 | .9487 |
| Lokalalei 2C (+2A, 2D) ↔ Nasura NAS3 | .7570 | .9439 |
| Nasura NAS3 ↔ Swartkrans Member 1 | .6927 | .8694 |
| Swartkrans Member 1 ↔ Gona DAN-2 | .7426 | .8772 |
| Gona DAN-2 ↔ Sterkfontein Member 5 | .6995 | .8772 |
| Sterkfontein Member 5 ↔ Kanjera South ( KS1-3) | In the main analysis | |
| Kanjera South ( KS1-3) ↔ Olduvai Gorge L.68 | .9727 | .8779 |
| Olduvai Gorge L.68 ↔ Drimolen Main Quarry | .9973 | .9917 |
| Drimolen Main Quarry ↔ Gona OGS-3 | .8105 | .3909 |
| Gona OGS-3 ↔ Ileret FwJj 20 (A 41) | Same central age | |
| Ileret FwJj 20 (A 41) ↔ Fejej FJ-1a | Same central age | |
| Fejej FJ-1a ↔ Ain Boucherit Ab-Up | .5440 | .5642 |
| Ain Boucherit Ab-Up ↔ Konso KGA6-A1 | .8711 | .8219 |
| Konso KGA6-A1 ↔ Kromdraai B | .4408 | .5325 |
| Kromdraai B ↔ Koobi Fora FxJj1 105 | .9060 | .9394 |
| Koobi Fora FxJj1 105 ↔ Olduvai Gorge DK | .9796 | .8234 |
| Olduvai Gorge DK ↔ Wonderwerk Stratum 12 | .5799 | .8190 |
| Wonderwerk Stratum 12 ↔ Olduvai Gorge FLK Zinj | .8905 | .9143 |
| Olduvai Gorge FLK Zinj ↔ Kokiselei KS 5,6 | .3479 | .3611 |
| Kokiselei KS 5,6 ↔ Kilombe GqJh13A,12B | .7283 | .5461 |
| Kilombe GqJh13A,12B ↔ El Beidha Levels A-C | .8630 | .8115 |
| El Beidha Levels A-C ↔ Naiyena Engol NY1 | .7634 | .6955 |
| Naiyena Engol NY1 ↔ Kalokodo 6 | Same central age | |
| Kalokodo 6 ↔ Olduvai Gorge HWK E | .8466 | .8842 |
| Olduvai Gorge HWK E ↔ Melka Kun. Garba IVE,IVF | .5657 | - |
| Melka Kun. Garba IVE,IVF ↔ Ain Hanech Levels A-C | .6553 | - |
| Ain Hanech Levels A-C ↔ Sare-Abururu SR-2 | Same central age | |
| Sare-Abururu SR-2 ↔ Melka Kun. Kella III | .5168 | - |
